# Airway foreign body removal in children: warning about shortcuts

**DOI:** 10.3389/fmed.2026.1928242

**Published:** 2026-10-14

**Authors:** Maria Majori, Alessandro Marchioni

**Affiliations:** 1 Pulmonology and Thoracic Endoscopy Unit, Cardiothoracic and Vascular Department, University Hospital of Parma, Parma, Italy; 2 Respiratory Diseases Unit, Department of Medical and Surgical Sciences, University of Modena Reggio Emilia, University Hospital of Modena, Modena, Italy

**Keywords:** flexible bronchoscope, foreign bodies, pediatric airway occlusion, respiratory failure, rigid bronchoscopy

Foreign body aspiration remains one of the most life-threatening emergencies in pediatric airway management, requiring prompt intervention to prevent rapid progression to severe hypoxemia. Although rigid bronchoscopy continues to be recognized as the gold standard for the removal of airway foreign bodies in children, recent technological advances have expanded the role of flexible bronchoscopy, fostering an ongoing debate regarding the optimal therapeutic approach. In this Perspective, we argue that this discussion should move beyond a simplistic comparison between rigid and flexible techniques. Instead, the primary distinction lies between clinically stable patients with distally located foreign bodies and life-threatening central airway obstructions, in which rigid bronchoscopy remains indispensable. We critically examine the current evidence supporting flexible bronchoscopic techniques, including the use of disposable bronchoscopes and cryotechnology, highlighting their potential advantages as well as their intrinsic mechanical and clinical limitations in pediatric patients. We further discuss the growing concern that the increasing reliance on flexible bronchoscopy may reflect a progressive loss of expertise in pediatric rigid bronchoscopy rather than a true evolution of standards of care. Ultimately, technological innovation should complement, rather than replace, proficiency in rigid airway management. Ensuring immediate availability of both techniques within appropriately trained multidisciplinary teams remains essential to guarantee the highest standards of safety for children with airway foreign body aspiration.

Although relatively uncommon, inhaled foreign bodies (IFBs) represent one of the most life-threatening emergencies in pediatric airway management, predominantly affecting children aged 1–2 years (1, 2). At this age, the millimetric caliber of the airways predisposes aspirated objects to cause the occlusion of the trachea or main bronchi. Most of the IFBs in pediatric patients, moreover, consist of organic, food-related materials prone to hygroscopic expansion, facilitating the progression from partial to complete airway obstruction. Furthermore, patients in this cohort exhibit heightened vagal sensitivity, predisposing them to laryngospasm and bradycardia. These physiological factors, combined with high oxygen consumption rates and reduced functional residual capacity (FRC), significantly accelerate the onset of hypoxia (3). In addition, the small caliber of the pediatric airway implies that even minimal reductions in lumen result in exponential increases in airflow resistance, as described by Poiseuille's law. The increased compliance of the pediatric chest wall further reduces airway support, promoting dynamic airway collapse during forceful inspiratory efforts, particularly in the setting of obstruction (Figure 1). Consequently, prompt and effective intervention is imperative, as fatal outcomes may rapidly occur due to severe hypoxemia.

**Figure 1:**
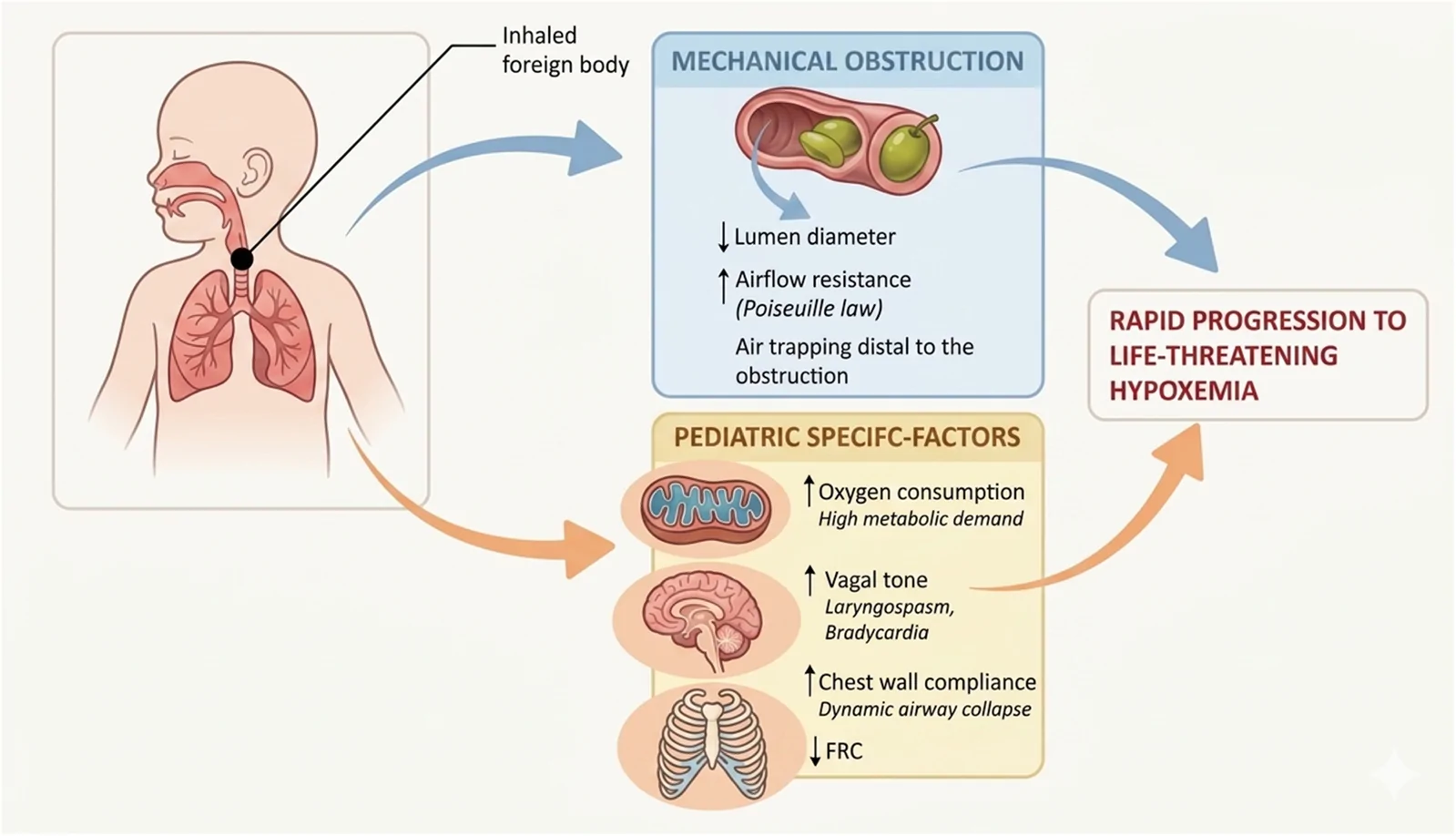
Pathophysiological cascade leading to severe hypoxemia in pediatric airway foreign body obstruction. Inhaled foreign bodies may cause acute mechanical airway obstruction, leading to a marked increase in airflow resistance and distal air trapping. In children, this effect is amplified by specific physiological features, such as increased oxygen consumption, heightened vagal tone, increased chest wall compliance with dynamic airway collapse and reduced functional residual capacity (FRC). The combinations of these factors result in a rapid progression to life-threatening hypoxemia.

Despite consensus statements and the prevailing literature identifying the rigid bronchoscope as the gold standard for safe and effective FB removal in children, the last decade has seen a proliferation of alternative techniques utilizing flexible bronchoscopy. Beyond considerations of healthcare costs and hospital stay duration, this shift is fundamentally driven by the limited widespread proficiency in pediatric rigid bronchoscopy. However, although flexible bronchoscopy might be indicated in selected, non-urgent cases, it does not address the life-threatening airway emergencies that constitute the primary focus of this clinical controversy.

When evaluating this issue, we must therefore first establish a clear distinction between two clinical scenarios: the management of obstructing foreign bodies (FBs) within the trachea or main bronchi -whose dimensions may preclude extraction through the rigid bronchoscope, requiring *en bloc* removal of the instrument and the FB via high-grip forceps- versus smaller FBs localized in the lobar bronchi, which may be safely retrieved via flexible bronchoscope through an endotracheal tube (ETT) or laryngeal mask airway (LMA) with minimal risk of impaction at the cricoid level. Beyond the mechanical aspects of foreign body retrieval, the physiological management of the shared airway deserves equal consideration. During pediatric airway endoscopy, maintenance of adequate ventilation and oxygenation is often as critical as the extraction itself. Rigid bronchoscopy provides a stable airway conduit that allows continuous ventilation while instruments are manipulated, thereby facilitating gas exchange and anesthetic control throughout the procedure. By contrast, flexible bronchoscopy performed through an endotracheal tube or laryngeal mask inevitably reduces the available airway lumen. In small children, this may significantly increase airway resistance and limit effective ventilation during prolonged manipulation, particularly if repeated extraction attempts become necessary. These physiological considerations further reinforce the role of rigid bronchoscopy as the preferred strategy whenever airway patency or respiratory stability may be compromised.

In recent years, the discourse has devolved into a confrontation between extraction techniques—rigid versus flexible bronchoscopy—a sterile debate that nonetheless invariably emphasizes the necessity of converting from a flexible to a rigid approach should the clinical situation deteriorate. Does this assertion not constitute an inherent contradiction? Does it not implicitly acknowledge the requirement for both instruments to be immediately available, necessitating the proficiency to operate both within an appropriate clinical setting? Ultimately, does this not underscore that, for the safety of the pediatric patient, these two modalities must be viewed as fundamentally complementary rather than mutually exclusive? Notably, available clinical data suggest that this need for conversion is far from anecdotal. In retrospective series, up to one-third of cases initially managed with flexible bronchoscopy ultimately require conversion to rigid bronchoscopy, exposing the patient to a second procedural step that may occur under less controlled and potentially unstable conditions (4). Furthermore, while overall complication rates between flexible and rigid bronchoscopy appear comparable, respiratory complications have been reported to occur less frequently during rigid procedures, highlighting a potential safety advantage in critical airway management scenarios (5). These findings challenge the notion that flexible bronchoscopy can be safely considered a primary strategy in all cases. Beyond the technical debate, a broader concern emerges regarding the progressive erosion of expertise in pediatric rigid bronchoscopy. In many centers, the increasing reliance on flexible techniques is not solely driven by clinical reasoning, but also by the declining availability of operators trained in rigid airway management. This trend raises a critical question: can a technique be considered a valid alternative when its adoption is primarily dictated by limitations in expertise rather than by patient-centered indications? Airway foreign body removal in children should not be framed as a choice between tools, but rather as a question of preparedness. The availability of both rigid and flexible bronchoscopy, combined with the ability to seamlessly transition between them, represents the true standard of care (6, 7). In this context, the concept of “complementarity” should not justify shortcuts, but instead reinforce the need for comprehensive training and appropriate clinical settings.

Undeniably, flexible bronchoscopy instrumentation has advanced considerably since Martinot et al. first restricted its role to diagnostic verification in clinically stable, non-asphyxiated patients with non-radiopaque foreign bodies (FBs). More recently, the evolution of flexible bronchoscopy has been further shaped by the introduction of single-use, high-definition video bronchoscopes, which combine improved optical performance with streamlined procedural logistics, obviating the need for dedicated video towers and reducing the operational footprint in the operating room. Their disposable design also mitigates concerns regarding damage to reusable instruments, particularly in technically demanding settings. This shift towards integrated, single-use platforms—now exemplified by the latest generation of bronchoscopes, reflects a broader trend towards accessibility and procedural adaptability in airway endoscopy. From a technical perspective, the combination of reduced outer diameter and relatively large working channels allows their use even in small pediatric airways, including insertion through narrow rigid bronchoscopes typically employed in early childhood. This hybrid approach may facilitate access to distal bronchial segments while maintaining airway control. However, despite these technological advances, a fundamental mechanical constraint remains: when an obstructing FB exceeds the internal diameter of the endotracheal tube, the flexible forceps must be maneuvered outside the conduit. This configuration significantly increases the risk of subglottic impaction or accidental loss of the foreign body during extraction, particularly when the object exceeds the internal diameter of the airway conduit. Beyond providing superior gripping force, the rigid bronchoscope also serves as a protective conduit during withdrawal, reducing direct contact between the foreign body and the vulnerable pediatric subglottic airway and thereby minimizing the risk of mucosal injury or complete airway obstruction. The emergence of nitinol baskets and cryoprobes has undoubtedly expanded the technical scope for flexible extraction. Nevertheless, these technological advances do not overcome the fundamental mechanical limitations discussed above.

To enhance the security of the grasp during flexible bronchoscopy, some authors advocate for the use of cryoprobes. Indeed, the 2019 introduction of the 1.1 mm cryoprobe, compatible with the 1.2 mm working channel of the smallest pediatric bronchoscopes, has significantly broadened the scope of cryotechnology in this field. However, while this adjunct offers undeniable advantages in adult patients, the pediatric context presents a different risk profile: given the constraints of pediatric airway, the potential for iatrogenic tracheobronchial mucosal injury is disproportionately high. Furthermore, the clinical utility of cryotechnology is inherently limited by the nature of the foreign body itself: it remains ineffective for inorganic materials, which lack the requisite water content for successful cryo-adhesion. Finally, adhesion forces may be limited in certain conditions. Regardless, it should be noted that current literature does not provide sufficient evidence to support this approach. For instance, in the largest clinical series, comprising 50 patients (8), only 24 were under two years of age, and specific data regarding this subgroup are lacking. In particular, while the overall success rate was reported at 94%, the study does not specify the success rate for the pediatric cohort, the precise anatomical location of the foreign body, or the number of attempts required to complete the procedure -noting that three cases were unsuccessful and two required multiple attempts. In the second largest cohort (9), comprising 16 patients with a mean age of 2.1 years, the procedural success rate was 69%. The case series by Zhang et al. (10) is even smaller, involving seven patients under the age of two, with a procedural success rate of only 71%.

Special consideration must be given to the aspiration of nuts, particularly peanuts, during early infancy. This issue is increasingly relevant following pediatric recommendations for early dietary introduction to prevent sensitization in adulthood (11). Unfortunately, such guidelines are not always accompanied by the essential clarification that these foods must be administered exclusively as purees or similar age-appropriate textures, rather than in their whole form. Once in contact with the airway mucosa, peanuts release oils that trigger extensive granulomatous reactions; these can encapsulate the foreign body, significantly complicating extraction and carrying an inherent risk of hemorrhage. Also in such challenging clinical scenarios, the use of a rigid bronchoscope remains mandatory. Organic foreign bodies, particularly peanuts, frequently induce marked mucosal inflammation and airway hyperreactivity, making repeated instrumentation potentially more hazardous. Under these circumstances, the ability to maintain secure airway control and optimal ventilation becomes a critical component of procedural safety. Ultimately, in pediatric airway emergencies, safety is not defined by the instrument alone, but by the expertise, preparedness, and multidisciplinary coordination of the team. This shift risks redefining standards of care based on feasibility rather than on safety.
